# *Paecilomyces*/*Purpureocillium* Infection in Children, Case Report, and Review of the Literature

**DOI:** 10.3390/jof8090930

**Published:** 2022-09-01

**Authors:** Musaed Alharbi, Nourah Alruqaie, Ahmed Alzahrani, Maha Almuneef

**Affiliations:** 1 Department of Pediatric Infectious Diseases, King Abdullah Specialist Children Hospital, Ministry of National Guard-Health Affairs (NGHA), Riyadh P.O. Box. 22490, Saudi Arabia; 2 King Abdullah International Medical Research Centre, King Abdulaziz Medical City, Ministry of National Guard-Health Affairs (NGHA), Riyadh P.O. Box. 22490, Saudi Arabia; 3 College of Medicine, King Saud bin Abdul-Aziz University for Health Science, Ministry of National Guard-Health Affairs (NGHA), Riyadh P.O. Box. 22490, Saudi Arabia; 4 Department of Medical Imaging, King Abdullah Specialist Children Hospital, Ministry of National Guard-Health Affairs (NGHA), Riyadh P.O. Box. 22490, Saudi Arabia

**Keywords:** *Paecilomyces*, *Purpureocillium*, fungal, osteomyelitis, pediatric, immunocompetent

## Abstract

*Paecilomyces*/*Purpureocillium* has recently been recognized as an emerging human pathogen, causing serious infection in immunocompromised and immunocompetent patients. Several predisposing factors have been reported, including foreign body implants, previous surgery, or trauma. Treatment with antifungal drugs often fails as species-specific differences in antifungal susceptibilities are one of the management challenges. Surgical debridement with or without antifungal therapy was sufficient to cure the infection in a few reported cases. Nonetheless, the surgical approach has been found to decrease the chance of dissemination and recurrence. Here, we report the first pediatric patient with chronic osteomyelitis of the femur secondary to *Paecilomyces* species, with no predisposing risk factors. Our case was successfully treated with a combination of antifungal therapy and surgical debridement. Additionally, we describe the first extensive literature review of previously reported *Paecilomyces*/*Purpureocillium* species infections in pediatric age groups.

## 1. Introduction

Fungal infections are well known to cause opportunistic infections, especially among immunocompromised patients, leading to increased morbidity and mortality [1,2,3]. Species of *Paecilomyces*/*Purpureocillium* are common environmental molds and are seldom associated with human infections. However, *Paecilomyces variotii* and *lilacinus* are considered emerging causative agents in immunocompromised patients and patients with underlying chronic diseases. *Paecilomyces lilacinus*, a common species reported clinically, had different morphological and phylogenetic characteristics; hence, Luangsa-Ard et al. had modified the name recently to *Purpureocillium lilacinum* [4].

*Paecilomyces*/*Purpureocillium* species can infect patients via foreign bodies, implanted devices like prosthetic valves, lens implants, catheters, ventriculoperitoneal shunts, or after trauma [2,3,4,5].

We describe the first case of an immunocompetent child with no underlying disease or predisposing factors presenting with chronic osteomyelitis due to *Paecilomyces*. Furthermore, we report the first literature review of *Paecilomyces*/*Purpureocillium* infection in the pediatric population, describing demographics, risk factors, diagnosis, treatment regimen, and outcomes.

## 2. Case

A previously healthy 12-year-old girl from a small village in Saudi Arabia presented to the emergency department in King Abdullah Specialized Children’s Hospital in Riyadh with left hip pain for five months. The pain was described as progressive, worsening while walking, and affecting her daily life. She had no history of trauma, fever, weight loss, raw milk ingestion, animal contact, or gastrointestinal or other systemic symptoms.

Upon physical examination, there was a limitation in left hip flexion, leg extension, external and internal rotation, as well as a leg-length discrepancy with an antalgic gait. The remainder of the examination was unremarkable.

Her laboratory investigations showed the following: white blood cells 5 × 10^9^/L (4.5–13.5 × 10^9^/L), hemoglobin 136 g/L (120–160 g/L), ESR 56 mm/h (0–22 mm/h), and CRP 7 mg/L (0–3 mg/L). Multiple sets of blood cultures were negative. As brucellosis is an endemic disease in Saudi Arabia, *Brucella abortus* and *Brucella melitensis* standard agglutination tests (SAT) were done with a titer of <1:160 (negative).

**Figure 1:**
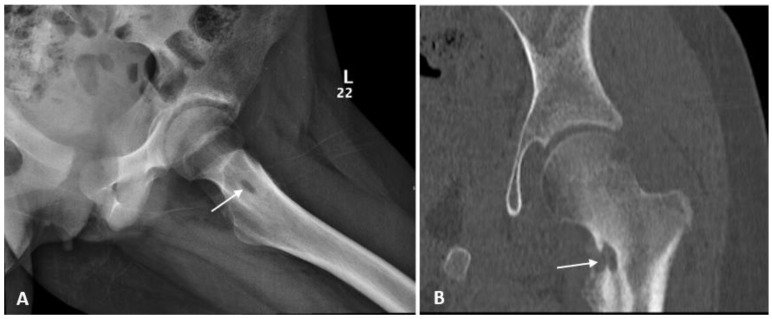
(**A**) Hip X-ray showed increased sclerosis with central lucency at the medial aspect of left femoral neck associated with the cortical irregularity and periosteal reaction; (**B**) Computed Tomography (CT) scan confirmed the erosive nature of the lesion with adjacent sclerosis and the adjacent bony overgrowth.

Non-specific arthritis was the preliminary diagnosis based on clinical, laboratory, and radiological findings of narrowing joint space. Follow-up plain radiograph of the affected hip showed sclerosis with central lucency at the medial aspect of the left femoral neck associated with cortical irregularity and periosteal reaction. Further evaluation by Computed Tomography (CT) scan revealed similar findings (Figure 1).

**Figure 2:**
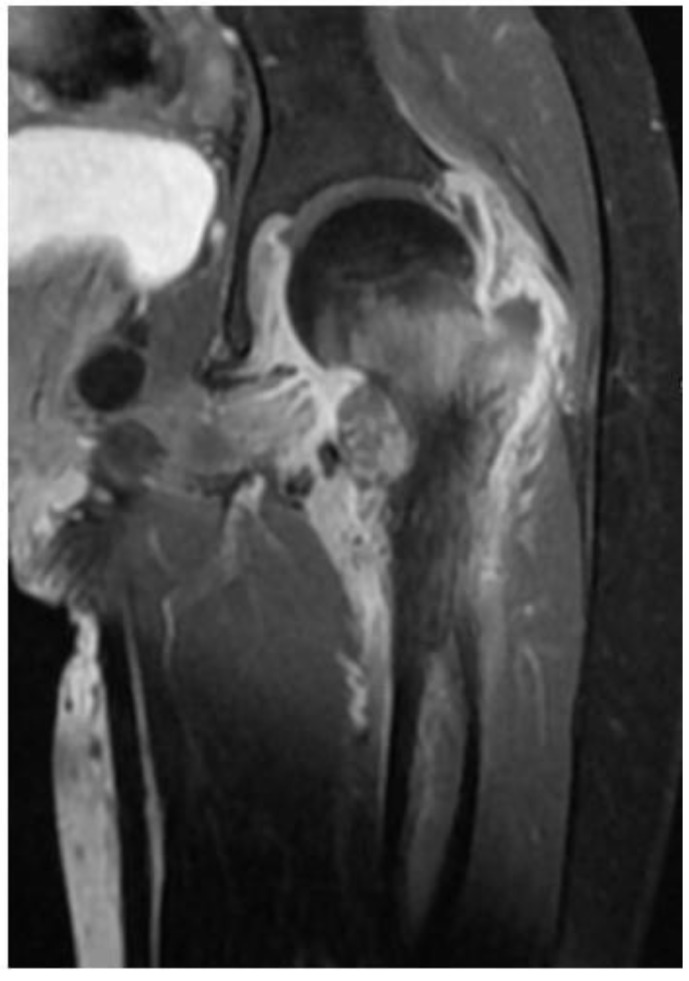
Magnetic Resonance Imaging (MRI) of the hip revealed bony overgrowth of the lesser trochanter with early cystic formation and a fluid-fluid level, extensive bone marrow edema, extensive adjacent soft tissue edema and hip joint effusion with evidence of synovial thickening and enhancement post contrast administration.

The patient was referred to the rheumatology service for further evaluation. Given the initial CT findings, Magnetic Resonance Imaging (MRI) of the hip was obtained, which showed evidence of chronic arthritis (Figure 2). Therefore, she was started on non-steroidal anti-inflammatory drug (NSAID) and given a working diagnosis of juvenile idiopathic arthritis (JIA).

A follow-up after one month of therapy revealed no clinical improvement. Hence, a percutaneous tissue (bone) biopsy was performed after the findings of worsening femur cortical lucent lesion with a sinus tract and extensive cortical thickening on the repeated CT scan.

**Figure 3:**
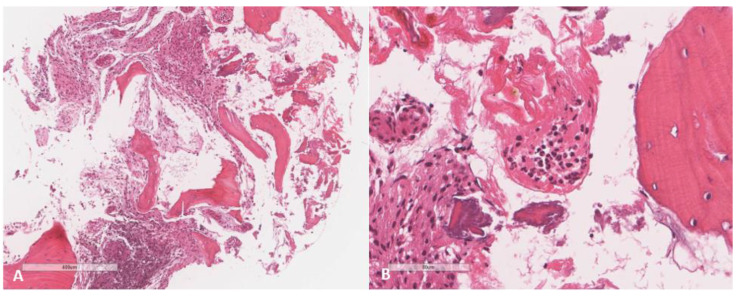
Histopathological findings in bone tissue (**A**) There are scattered bone trabeculae and fibroconnective tissue exhibiting dense inflammation and collection of hemosiderin-laden macrophages (lower left); (**B**) Collection of neutrophils forming micro-abscess.

The tissue samples for bacterial, fungal, and acid-fast bacilli cultures were negative. Although there were no findings suggestive of infection or malignancy in the histopathological examination, an ongoing inflammatory process was noted. Therefore, she continued on NSAIDs. At a three-month follow up, her clinical condition remained the same with no significant improvement. Thus, she was referred to pediatric infectious diseases where she was diagnosed with chronic osteomyelitis. The repeated MRI revealed an increase in the size of the cortical lesion, breaking through the cortex and extending into the joint capsule with bone marrow edema. Consequently, a core biopsy was done to increase the yield of diagnostics. Multiple bone tissue cultures showed a growth of yellowish velvety texture colonies on Sabouraud dextrose agar, identified as *Paecilomyces* species by matrix-assisted laser desorption ionization time of flight mass spectrometry (MALDI-TOF). Polymerase chain reaction (PCR), and identification of the isolate to the species level along with susceptibility testing were not performed due to a limitation of laboratory resources. However, based on the morphological appearance of the culture, it was identified as *Paecilomyces variotii*, histopathologically showing fragments of necrotic bone and fibroconnective tissue with heavy inflammation and abscess formation, with no fungal elements on Grocott methenamine silver (GMS) stain (Figure 3). In contrast to intravenous options such as amphotericin B, and unavailability of posaconazole in our center, voriconazole had been chosen to be the initial antifungal therapy as it was previously reported to be effective in treating this organism, along with the availability as an oral formulation and capability to monitor serum therapeutic level.

**Figure 4:**
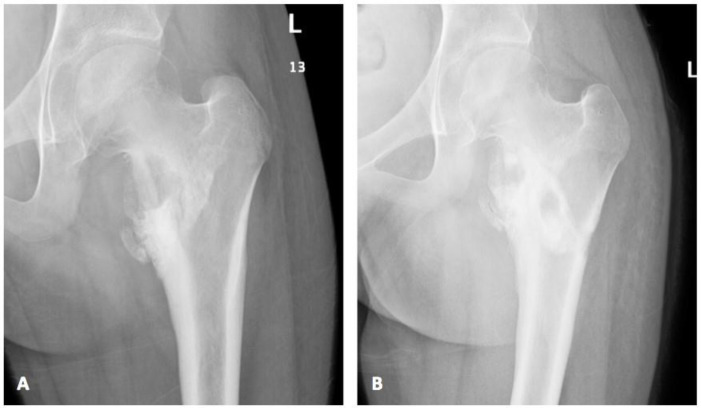
(**A**) Pre-surgical X-ray showed worsening of medial femoral erosions and destruction with adjacent sclerosis; (**B**) Post-surgical X-ray demonstrated interval sclerosis and healing with new bone formation along the medial aspect of femoral neck with interval reduction of erosive changes and medial neck lytic lesion.

Despite three months of therapy on voriconazole (9 mg/kg/dose twice daily) and appropriate therapeutic level between 2.5–4.18 mcg/mL, her clinical status remained unchanged. Thus, a surgical debridement with extensive washout and debris removal was performed. In spite of negative tissue culture this time, which might have resulted from the three months of prior antifungal treatment, similar histopathological findings of inflammation and micro-abscess appearance were noted. One-month post-surgical intervention, a remarkable clinical and radiological improvement was noted (Figure 4). Post-operatively, she completed a total duration of 12 weeks of therapeutic voriconazole with an appropriate trough serum level and all subsequent follow-up visits showed complete clinical recovery and no evidence of recurrence.

Given the reported immune defects associated with this type of infection, all the immunological workups, including primary immunodeficiency panel, neutrophil oxidative burst test, and immunoglobulins levels, were completely normal.

## 3. Discussion

*Paecilomyces/Purpureocillium* are saprophytic fungi found in air, soil, and decaying plants. It is known for its resistance to most sterilizing techniques, making the chance of contamination very high. *Paecilomyces* genus has several species. A few of them, such as *P. lilacinus*, *P. variotii*, *P. marquandii* and *P. javanicus*, have been described as pathogenic in humans [1,6,7,8].

Microscopically, *Paecilomyces*/*Purpureocillium* are described by their swollen phialides at their base that taper gradually toward the neck and long ovoid conidia chains attached to the tips. Morphologically, they are well known by their fluffy powdery texture. However, colonies can show different colors, depending on the type of species. Examples are the yellow-brown of *P. variotii* and the lilac of *P. lilacinus* [7,8,9].

Identification of *Paecilomyces* in clinical practice is crucial, however, due to similarities with other fungi, identification difficulties have been reported. *Paecilomyces* and *Penicillium* have similarities in reproductive structures, leading to difficulty in identification without specialized optic and measuring devices that are available in highly specialized reference labs. *Paecilomyces* can be identified effectively by molecular targeting internal transcribed spacer (ITS) regions within the ribosomal deoxyribonucleic acid (rDNA) [10].

We conducted a literature review of all English-language reports using PubMed and Embase database up to May 2021, using the keywords ‘*Paecilomyces’*, ‘pediatric’, and ‘osteomyelitis’. The literature reveals 30 cases, apart from our own, whose main characteristics are described in (Table 1).

Older children are frequently affected, as we found that 22 out of 30 (73%) reported cases were older than five years. *P. lilacinus* 12/30 (40%) and *P. variotii* 11/30 (36%) were the most commonly reported pathogenic species.

Remarkably, the majority of cases were catheter-related infections (either central venous line or dialysis catheter) (11/30 (36%)), followed by cutaneous lesions (8/30 (26%)). Sinopulmonary infections were also seen in (6/30 (20%)) cases. Additionally, few cases were reported involving eyes, lymph node, and skin and soft tissue, making a specific site predilection for this organism hard to be determined. Although there is a previously reported case of osteomyelitis in an 18-year-old male with an underlying immunodeficiency (chronic granulomatous disease) [19], our patient is the first case with no underlying medical condition and no predisposing risk factors. It is not clear whether our patient developed osteomyelitis secondary to transient unnoticed fungemia or spontaneous infection.

In the previous reports, patients with decreased immunity (primary or secondary) were commonly affected. Chronic granulomatous disease (CGD), blood malignancy, and prolonged neutropenia after bone marrow/solid organ transplant patients were found in most of the patients [12,14,16,17,19,20,21,22,23,27,28,29,33], and although infection in immunocompetent patients has been also reported, several predisposing risk factors are commonly present [11,18,24,25,30,32,35,36,37,38,40].

Although resolution was the usual outcome of this infection with no reported recurrences, death was seen in 4/30 (13%) patients [12,21,23]. While Das et al. have reported a fatality case due to bronchopneumonia, bronchoalveolar lavage (BAL) revealed two additional *Aspergillus* species, making the attribution of death secondary to *Paecilomyces*/*Purpureocillium* species alone is difficult [26]. Noticeably, all deaths related to *Paecilomyces*/*Purpureocillium* infection had an initial cutaneous lesion and then disseminated to the lungs or were complicated by graft-versus-host-disease (GVHD), which signifies the risk of dissemination.

The treatment options for *Paecilomyces*/*Purpureocillium* infection are challenging. Identifying the *Paecilomyces*/*Purpureocillium* at the species level is crucial due to the diversity of antifungal susceptibility of each species. Previous in-vitro studies reported high amphotericin B, itraconazole, flucytosine, and echinocandins minimum inhibitory concentrations (MICs) to *P. lilacinus*, which contributed to a high rate of treatment failure [3,41]. However, in-vitro activity of newer antifungals (i.e., Posaconazole) showed a good pattern of efficacy, considering it a drug of choice for *P. lilacinus* along with voriconazole and terbinafine [42,43,44,45,46]. In contrast, in-vitro susceptibilities of *P. variotii* species to amphotericin B, echinocandins, and most of the azole group, including posaconazole and itraconazole (excluding fluconazole), had been well documented, making them an available option for therapy. However, amphotericin B is still considered the drug of choice for this species [47].

Voriconazole, on the other hand, has variable activity against *P. variotii*. Although the resistance had been reported in some studies [28,42,43,44,45,46], a successful treatment with voriconazole had also been reported [3,34], making testing this drug susceptibility a valuable step in management. Combination therapy and synergism have not been fully evaluated clinically. However, Ortoneda et al. reported that the best synergistic interaction for *P. lilacinus* was a combination of terbinafine and voriconazole, whereas combining terbinafine with itraconazole was successful for treating *P. variotii* [48]. While in vivo, Toker et al. and Chang et al. reported two cases with clinical improvement and resolution of infection after combining voriconazole with terbinafine [31,36].

The removal of prosthesis/catheters and surgical debridement along with antifungal therapy are essential for an optimal cure and will decrease the risk of complication or recurrence. Lazarus et al. and Pastor et al. found an increased failure rate in patients treated with medication solely without the removal of catheters or surgical debridement [1,49]. Hence, surgical source control is of crucial importance.

Unfortunately, in our case, we could not identify the isolate to the species level due to a limitation of laboratory resources and the inability to send it to the central laboratory due to the COVID-19 pandemic. However, it is crucial to acquire species identification for any rare fungi, as antifungal susceptibility between different species is variable and it plays a major role in the management. In chronic osteomyelitis, particularly fungal osteomyelitis, it is of the utmost importance that pediatric infectious diseases service is involved early in the management process in order to provide appropriate diagnostics and therapeutics while improving patient outcome. In our case, a combination of surgical debridement and antifungal therapy resulted in a successful outcome, highlighting the importance of surgical intervention in chronic osteomyelitis.

## 4. Conclusions

*Paecilomyces*/*Purpureocillium* infection, although commonly thought to be a contaminant, is a rare but potentially serious infection with severe complications and tendency to dissemination. This is the first reported case in an immunocompetent child with no predisposing risk factors. The management strategy is universally challenging; however, the key to a successful treatment is surgical intervention, if feasible, along with antifungal therapy with or without synergism.

## Figures and Tables

**Table 1:** *Paecilomyces*/*Purpureocillium* Infections in Pediatrics.

Report	Age/Gender	Underlying Condition	Diagnosis	Site of Culture	Organism	Management	Outcome
Rodrigues et al. [11]	17Y/M	S/P trauma by large nail from barn	Endophthalmitis	Corneal scraping	*P. viridis*	Pimaricin topical ^†^ Enucleation	Enucleation of the eye
Murciano et al. [12]	6Y/F	ALL, prolong neutropenia on chemotherapy	Cutaneous lesions disseminated to lungs	Skin biopsy	*P. lilacinus*	AMB plus 5-FC ^†^	Death
Crompton et al. [13]	13Y/M	Bilateral Wilms tumor, S/P resection, chemoradiation, on PD	Peritonitis	Peritoneal fluid	*P. variotii*	Removal of catheter AMB until clinical improvement, then discharged on FLC for 4 weeks	Resolution
Williamson et al. [14]	8Y/M	CGD	Purulent cellulitis	Culture from debridement	*P. variotii*	AMB for 7 weeks, then ITC for 1 year	Resolution
Tan TQ et al. [15]	18mo/M	Rhabdomyosarcoma on chemotherapy	Catheter-related fungemia	Central line blood culture	*P. lilacinus*	Removal of port-A-cathAMB for short course ^†^	Resolution
Silliman et al. [16]	4Y/M	CGD	Abdominal wall abscess	Fine needle aspirate	*P. lilacinus*	AMB for 2 months	Resolution
Bernacer et al. [17]	7Y/M	ALL, neutropenia on chemotherapy	Catheter-related fungemia	Central line blood culture	*P. lilacinus*	Removal of central line AMB ^†^	Resolution
Marzec et al. [18]	12Y/M	ESRD, on PD	Peritonitis	Peritoneal fluid	*P. variotii*	-Failed the initial treatment with intraperitoneal AMB for 10 days -Catheter was removed and symptoms resolved without further antifungal therapy	Resolution
Cohen-Abbo et al. [19]	18Y/M	CGD	Multifocal osteomyelitis, pneumonia	Bone and lung biopsy	*P. variotii*	Total dose of AMB, IFN-γ, then ITC for 1 year	Resolution
Shing et al. [20]	NM	S/P bone marrow transplant	Catheter-related fungemia	Central line blood culture	*P. variotii*	Removal of central line AMB plus ITC for 3 months	Resolution
Orth et al. [21]	14Y/F	AML, S/P bone marrow transplant, GVHD	Disseminated necrotizing skin eruption	Skin biopsy	*P. lilacinus*	AMB, ITC, FLC, GSV, TBF, 5-FC ^†^	Death
Smitt et al. [22]	12Y/M	CGD	Pneumonia	Lung biopsy	*P. species*	AMB for 4 weeks, then ITC	Resolution
Itina et al. [23]	15Y/F	Hematological malignancy, S/P bone marrow transplant, GVHD, neutropenic	Cutaneous lesions	Skin swab	*P. lilacinus*	AMB, ITC, FLC, GSV, TBF, 5-FC ^†^	Death
Rinaldi et al. [24]	14mo/M	Congenital bilateral renal hypoplasia, on PD	Peritonitis	Peritoneal fluid	*P. variotii*	FLC IV and intraperitoneal for 4 weeks	Resolution
Nayak et al. [25]	8Y/M	Healthy child, S/P polypectomy	Sinusitis	Tissue from ethmoid, maxillary and sphenoid sinuses	*P. lilacinus*	Fronto-spheno-ethmoidectomy with maxillary clearance ITC for 6 months	Resolution
Das et al. [26]	9.5Y/F	CF, S/P bilateral lobar-lung transplant	Pneumonia	Bronchoalveolar lavage	*P. variotii* *A.fumigatus* *A. niger*	AMB ^†^	Death
Roque et al. [27]	5Y/M	AML, neutropenia on chemotherapy	Fungemia and cutaneous lesions	Blood and bone marrow cultures	*P. lilacinus*	AMB, FLC, and ITC ^†^	Resolution
Chamilos et al. [28]	14Y/M	ALL, on chemotherapy and steroids, prolong neutropenia On VRC as prophylaxis	Fungemia disseminated to lungs and skin	Blood culture Skin biopsy	*P. variotii*	Removal of central line AMB for 2 months Kept on ITC as prophylaxis	Resolution
Wang et al. [29]	21mo/M	CGD	Splenic abscess	Culture of the abscess	*P. variotii*	Partial splenectomy, FLC and 5-FC for total 14 months	Resolution
Jackson et al. [30]	14D/F	35 week gestation, down syndrome with NEC	Fungemia	Blood culture	*P. lilacinus*	AMB and discharged on FLC maintenance ^†^	Resolution
Chang et al. [31]	15Y/M	Reflux nephropathy, S/P bilateral nephrectomy and renal transplant complicated with rejection, on PD	Peritonitis	Peritoneal fluid	*P. lilacinus*	-Failed the initial treatment with AMB and FLC-Catheter was removed and switched to oral VRC with no improvement -Then added TBF every other day with VRC for three months	Resolution
Yuan et al. [32]	17Y/F	Extended-wear soft contact lens	Keratitis	Corneal scraping	*P. lilacinus*	Natamycin 5% topical ^†^	Resolution
Bogomolova et al. [33]	13Y/M	ALL, neutropenic	Invasive mycosis with destruction of the septal cartilage	Nasal swab culture, Histopathological findings from damaged cartilage	*P. lilacinus*	VRC for 80 days	Resolution
Polat et al. [34]	16Y/M	Wilson disease, S/P liver transplant, with peritoneal drainage	Peritonitis	Peritoneal fluid	*P. variotii*	AMB and VRC for 10 days Then discharged on VRC for 4 weeks	Resolution
Kuboi et al. [35]	6D/M	Premature 23-week gestation, part of twin	Cutaneous lesions	Skin culture	*P. formosus*	IV micafungin and topical lanoconazole for 22 days	Resolution
Toker et al. [36]	14Y/M	S/P keratoplasty	Keratitis	Corneal scraping	*P. species*	-Failed one week therapy on IV FLC and topical 2% FLC plus intracameral injection AMB and topical 0.3% AMB -Switched to TBF and topical 1% VRC and IV VRC ^†^	Resolution
Çolakoglu et al. [37]	14mo/M	Ureteropelvic obstruction, Symptomatic for 10 months since double –J (D-J) catheter was removed	Urinary tract infection	Tissue fragments from urine	*P. variotii*	AMB for 15 days	Resolution
Anand et al. [38]	15Y/F	S/P TOF repair and prosthetic pulmonary valve	Infective endocarditis	Tissue culture from pulmonary valve	*P. species*	Pulmonary valve excision IV AMB plus oral VRC ^†^	Resolution
Tiwari et al. [39]	3Y/M	Hodgkin’s lymphoma	Lymphadenopathy	Lymph node biopsy	*P. species*	VRC for 3 weeks	Resolution
Chen et al. [40]	11Y/F	Extended-wear soft contact lens	Keratitis	Corneal scraping	*P. species*	Ciprofloxacin ^†^	Resolution
Index case	12Y/F	Healthy child	Chronic osteomyelitis	Tissue culture	*P. species*	VRC total 6 months with surgical debridement	Resolution

**Notes**: S/P, status post; P, *Paecilomyces/Purpureocillium*; ALL, acute lymphocytic leukemia; AMB, amphotericin B; 5-FC, flucytosine; PD, peritoneal dialysis; FLC, fluconazole; CGD, chronic granulomatous disease; ITC, itraconazole; ESRD, end stage renal disease; IFN-γ, interferon gamma; GVHD, graft versus host disease; TBF, terbinafine; GSV, griseofulvin; AML, acute myeloid leukemia; CF, cystic fibrosis; A, aspergillus; VRC, voriconazole; NEC, necrotizing enterocolitis; TOF, tetralogy of Fallot; IV, intravenous. ^†^ Duration of therapy was not mentioned.

## Data Availability

Not applicable.
